# Modelling the interaction between wearable assistive devices and digital human models—A systematic review

**DOI:** 10.3389/fbioe.2022.1044275

**Published:** 2023-01-10

**Authors:** David Scherb, Sandro Wartzack, Jörg Miehling

**Affiliations:** Friedrich-Alexander-Universität Erlangen-Nürnberg, Engineering Design, Erlangen, Germany

**Keywords:** digital human model, musculoskeletal modelling, multi-body dynamic, interaction modelling, systematic review, soft tissue, wearable assistive device

## Abstract

Exoskeletons, orthoses, exosuits, assisting robots and such devices referred to as wearable assistive devices are devices designed to augment or protect the human body by applying and transmitting force. Due to the problems concerning cost- and time-consuming user tests, in addition to the possibility to test different configurations of a device, the avoidance of a prototype and many more advantages, digital human models become more and more popular for evaluating the effects of wearable assistive devices on humans. The key indicator for the efficiency of assistance is the interface between device and human, consisting mainly of the soft biological tissue. However, the soft biological tissue is mostly missing in digital human models due to their rigid body dynamics. Therefore, this systematic review aims to identify interaction modelling approaches between wearable assistive devices and digital human models and especially to study how the soft biological tissue is considered in the simulation. The review revealed four interaction modelling approaches, which differ in their accuracy to recreate the occurring interactions in reality. Furthermore, within these approaches there are some incorporating the appearing relative motion between device and human body due to the soft biological tissue in the simulation. The influence of the soft biological tissue on the force transmission due to energy absorption on the other side is not considered in any publication yet. Therefore, the development of an approach to integrate the viscoelastic behaviour of soft biological tissue in the digital human models could improve the design of the wearable assistive devices and thus increase its efficiency and efficacy.

## 1 Introduction

Exoskeletons, orthoses, exosuits, assisting robots and such devices are special products designed to support the human body in a specific way. These devices augment or protect the human body by applying force to it and are additionally wearable. Attempting to identify a generic term for these types of devices, in this publication the expression “wearable assistive devices” (WADs) following Asbeck et al. (2014) is used. In other publications, these product types are referred to as “physical assistive devices” (Mombaur and Ho Hoang, 2017), “wearable power assistive devices” (Imamura et al., 2011), “support systems/devices” (Miehling et al., 2018), “physical support systems” (Argubi-Wollesen and Weidner, 2018), “assistive devices” (Sartori et al., 2012) or just “exoskeletons” (Collins et al., 2015). Regarding their intended use, WADs can be classified into three groups (Young and Ferris, 2017). The first group are the devices that augment the performance of healthy subjects. First, they increase the humans’ strength and endurance and minimize the risk of injury (Tröster et al., 2020; Fritzsche et al., 2022), making them suitable in a working environment, for e.g., lifting objects (Millard et al., 2017) and working over-head (Molz et al., 2022). Second, the human effort for accomplishing activitites should be reduced. A common example for this application is the metabolic energy decrease for different activities (Collins et al., 2015; Dembia et al., 2017; Miehling et al., 2018). The second group of classification for WADs aims to support humans with an enduring disability. Motor or neurological disorders like stroke, spinal cord injury or cerebral palsy can lead to difficulties for affected subjects to execute movements. WADs aid people to perform movements they are unable to do on their own, like walking (Afschrift et al., 2014; Michaud et al., 2019; Yamamoto et al., 2019) or arm movements (Rahman et al., 2007). The third category are devices for therapeutic rehabilitation, which accounts for a specific crossover with the second group. These devices assist disabled humans after a disease until the previous performance of the body is restored (Shi, 2018; Akbas and Sulzer, 2019; Zhou et al., 2020). Therefore, the difference to the second group is represented by the temporary use of the products.

WADs need to provide sufficient support to the human body to be useful. By performing user tests, the efficacy of the devices can be evaluated and improved. Popular parameters investigated are the oxygen consumption or heart rate of the subjects to observe the physical load on the body (Danielsson and Sunnerhagen, 2004; Fritzsche et al., 2021) and user (dis-)comfort to analyze product acceptance and product safety (Mills et al., 2012; Lucas-Cuevas et al., 2014; Linnenberg and Weidner, 2022). Thus, user tests are beneficial to improve the design of the WAD and make it more suitable to users’ requirements. However, there are some problems with user tests. The steps of building a prototype, testing it on different users, gathering user feedback, adapting the design of the device, building a new prototype and thus starting the cycle from the beginning results in a very cost- and time-intensive process to design a final product (Agarwal et al., 2013; Ferrati et al., 2013; Fritzsche et al., 2021). Due to the interaction of WADs with the human body, it is also suitable to investigate the effects on the biomechanics of the users (Yamamoto et al., 2019). The biomechanical parameters, however, are mostly either hard to measure, e.g., using electromyography (EMG) to investigate muscle activations (Fritzsche et al., 2022; Molz et al., 2022) or in some cases even impossible to determine, e.g., joint reaction forces (Neptune et al., 2000; Zhou, 2020) during a study. Additionally, the use of WADs in user tests can be limited due to ethical and legal restrictions (Neptune et al., 2000; Fritzsche et al., 2022). Patient safety is the most critical aspect to consider, restricting the freedom of testing different properties of the device and requiring justification of the benefits of the test type. In order to address these challenges, the trend of using digital human models (DHMs), and more precisely musculoskeletal human models (MHMs) to evaluate WADs has emerged in recent years. Especially MHMs provide the advantages of investigating the effects on the human body itself, in particular on parameters of the musculoskskeletal human system like muscle activations and joint reaction forces, and evaluating the interactions between the human and the device design (Fournier et al., 2018) without compromising the health of the user. Further advantages of this tool include the elimination of the need to create prototypes in early design stages, the possibility to test different configurations of the device, with the effects of these variations being identified easily and more quickly, the early acquisition of more technical knowledge, a better performance and quality in the final product and, most importantly, a reduction in design time and cost (Agarwal et al., 2013; Ferrati et al., 2013; Zhou and Li, 2016; Dembia et al., 2017). However, all insights during the execution of user tests cannot be completely substituted by musculoskeletal simulation studies, which makes the simulation a good complementary tool to experimental testing (Manns et al., 2017).

**FIGURE 1:**
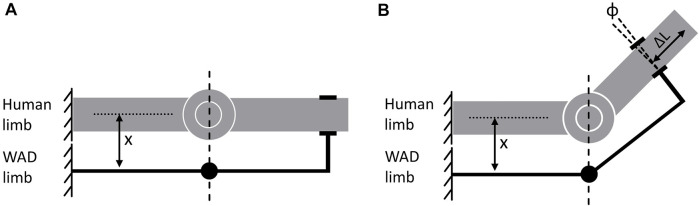
Misalignment shown on a simple degree of freedom joint attached with a WAD based on (Schiele and van der Helm, 2006); **(A)** initial aligment of human limb and WAD limb, **(B)** Occurring relative motion between human limb and WAD limb due to movement of the single degree of freedom joint of the human limb.

In order to build up a complete human-WAD model, a lot of elements have to be considered for modelling. The main goal is always the recreation of natural circumstances in the virtual representation to account for the correctness of simulation results and transferability to reality (Ferrati et al., 2013). Thus, elements like the device itself with its dimensions and material properties resulting in parameters like mass or inertia (Ferrati et al., 2013), the combination of single parts of the WAD, e.g., *via* joints (Millard et al., 2017), functional parameters of the devices [e.g., orthosis stiffness (Yamamoto et al., 2019)], controllers for regulation of the device (Durandau et al., 2019; Yin et al., 2019), actuation power and timing (Fournier et al., 2018) and many more elements, depending on the specific WAD itself, have to be included into the virtual environment. A key indicator for the efficient interaction between the human body and the device is the interface (Sánchez-Villamañán et al., 2019). The interface consists of the attachment types of the product (mostly straps or cuffs) and mainly of the biological soft tissue (skin, fat, muscles, etc.), which covers the movement-executing structure of the human body, the bones, which the WAD actually targets (Yandell et al., 2017; Young and Ferris, 2017). The biological soft tissue determines human comfort and possible occurring injuries, like scratches or bleedings (Young and Ferris, 2017). Ultimately, two main factors concerning the biological soft tissue influence the resulting efficiency and effectivity of the transmitted assistance by the device (Sánchez-Villamañán et al., 2019). The first one is the occurring relative motion between the human body and the device, resulting in the so-called misalignment (Figure 1). Due to the attachment of the WAD on the skin of the human, a distance (x) between the joint centers of both collaborators is present in reality (Figure 1A). Consequently, the movement of the human limb results in a shift Δ L and a rotation φ of the device (Figure 1B), which affects the efficacy of the support and can cause discomfort and pain for the users due to compressed and sheared skin (Schiele and van der Helm, 2006; Zanotto et al., 2015; van Dijk et al., 2017). Furthermore, the identification of the subject’s joint axes to perfectly align the WADs joint axes is very challenging, consequently resulting in the misalignment (Näf et al., 2018; Mallat et al., 2019). The second factor influencing assistance of a WAD is the tendency of the biological soft tissue to act as an energy sink (Young and Ferris, 2017). Yandell et al. (2017) demonstrated in their study that about 25% of the power provided by their device was lost during transmission. The remaining 75% indeed augmented the human body, but part of it was absorbed during loading and had therefore a delayed contribution to the assistance. Other contributions even report a power loss of 50% of the provided energy (Asbeck et al., 2015). Thus, the compliance and viscoelastic behaviour of the biological soft tissue can have a considerable influence, resulting in a desired support for the human body (Pons, 2010; Rossi et al., 2011; Quinlivan et al., 2016). Asbeck et al. (2014) are even indicating human-machine interface compliance as a major roadblock to designing exoskeletons.

Considering the rising use of DHMs for designing WADs, the biological soft tissue is missing in DHMs due to the underlying rigid body dynamics. This raises the question whether this important and non-negligible influence of the compliant biological soft tissue is taken into account. A missing consideration could lead to results that deviate from reality and, accordingly, to different or incorrect implications for the design and application of the device and, consequently, to negative effects for the user (Schiele and van der Helm, 2006; Zanotto et al., 2015). There should be different possibilities or approaches to model the interaction between human body and device, in order to ensure a valid transfer of the obtained information to reality. Thus, we want to investigate and answer the following research questions in this contribution:

RQ1: How is the interaction between wearable assistive devices and human represented by existing models in the digital simulation environment?

RQ2: How is the influence of the biological soft tissue at the interface between human and wearable assistive devices (regarding the occurring relative motion and alteration of power support) considered in these interaction models?

Our aim is to show different possibilities and approaches for modelling the interaction between DHMs and WADs, which was not done yet to our knowledge and to analyze these interaction modelling approaches in terms of their consideration of real-world interface behaviour. Furthermore, we wanted to investigate the consideration of the biological soft tissue in the digital human simulation of WADs, which is in our opinion a key factor for a sufficient transfer of gained simulation results to the design of a physical device.

## 2 Methods

### 2.1 Search strategy and study selection

To answer the aforementioned research questions, a systematic review of the literature was conducted. The electronic databases “Scopus” and “Web of Science” were searched. The basic search string resulted in a combination of, first, digital human models and synonyms and, second, WADs and other names of this product type (as already mentioned in the introduction) and names of single products like exoskeleton or orthosis. The used search string was: [(“musculoske* model*”) OR (“musculoske* simul*”) OR (“digital* human model*”) OR (“human model*”) OR (“biomech* model*”) OR (“biomech* simulat*”) OR (“computat* model*”) OR (“human neurom* model*”)] AND [(exoskelet*) OR (“assisti* device*”) OR (orthos?s) OR (orthotic*) OR (exosuit*) OR (“assist* robot*”) OR (“assist* robot* device*”) OR (“support* device*”)]. The specific search strings for each database can be found in the Supplementary Material (“Full search strings”). This generic string was chosen to find publications using multibody simulations of WADs and to identify the modelled interaction or interface between the model and the device. This strategy was applied to increase the probability of finding papers describing the modelled interaction or interface of digital human models and WADs, since “(interaction OR interface) modelling” is not explicitly mentioned in many papers. For the applied search string, all paper types published in English language were included in the systematic review. In “Web of Science” the literature search was conducted in all fields, whereas in “Scopus” the search was conducted on title, Abstract and Keywords. The date of the literature research was 18 March 2022. After finishing the analysis, an additional check of the databases was conducted on 15 August 2022 to identify published material in the meantime.

### 2.2 Literature scanning process

The found literature of every database was extracted and saved in Microsoft Excel (Microsoft, 2016). Then, a self-written macro was applied to identify and remove all duplicates. With the resulting publications, the real screening process began. First, the titles and abstracts of the identified publications were screened. In this step, mainly papers not using DHMs (e.g., finite-element models) and papers analyzing devices not embedded to the definition of WADs from the introduction (e.g., wheelchairs or hearing aids) were excluded. In the next steps, the remaining papers were screened by reviewing the material and methods and full paper. The material and methods part was especially screened because the type of modelled interaction or interface created is described in this part. During these two review loops, publications were excluded if they, first, were not dedicated to the interaction of DHMs and WADs, but rather depict the interface between the device and a computer to regulate the device (Fleischer and Hommel, 2008). Secondly, papers were excluded that used DHMs as a pre-investigating tool for the design of the WADs. For example, if investigations concerning the biomechanical changes of humans with a disease (e.g., after stroke or with crouch gait) were conducted to gain knowledge for a later use or design of WADs (Knarr et al., 2013; Steele et al., 2013; Waterval et al., 2021) or if DHMs were analysed to identify intervals for muscle activities or joint torques the device should support or replace (Durandau et al., 2019). The last exclusion criterion was the use of DHMs to evaluate effects of physical, manufactured WADs on a diseased patient (Ewing et al., 2016; Choi et al., 2017; Yamamoto et al., 2019). In this scenario, a patient is mostly equipped with a WAD, is recorded in a motion laboratory and the DHM is used to identify biomechanical data of the recorded data, like joint angles or torques (Yamamoto et al., 2019).Basically, papers were included in the literature review that simulated the effect of WADs on DHMs and therefore had to couple the two collaborators in the simulation. Additionally, the reference lists of the included publications were screened to identify important literature that were missed during the mentioned screening process and also included in the literature review. The full text of all included papers was then analyzed for eligibility and to identify and classify approaches for interaction/interface modelling between DHMs and WADs and to examine them regarding their strengths and limitations.

## 3 Results

### 3.1 Filtering *via* search strategy

**FIGURE 2:**
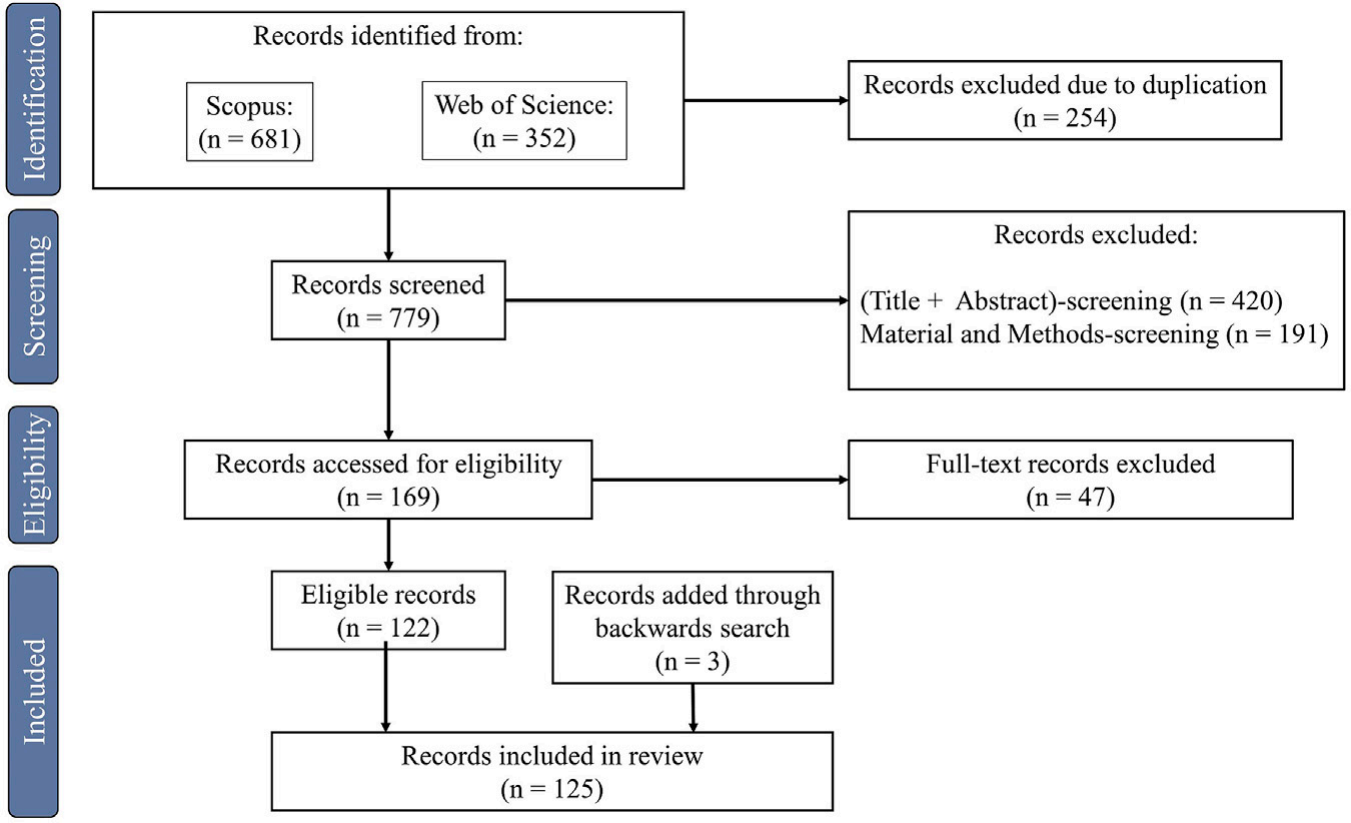
PRISMA flow diagram of study selection and screening process.

Figure 2 presents the study selection and screening process and the final outcome of included papers in the literature review. After searching all databases, 1,033 records were identified. 254 of these records could be removed due to duplications, resulting in 779 publications for screening. Papers were excluded during the screening process based on the aforementioned exclusion criteria. During screening of title and abstract 420 records and after screening of material and methods 191 records were excluded. The full text of the remaining publications was checked for eligibility of these records resulting in an exclusion of another 47 records. By the backward search in the reference lists of the remaining records, three papers were added to the literature review, resulting in 125 included publications that were further analyzed.

### 3.2 Interaction modelling approaches for DHMs and WADs

**FIGURE 3:**
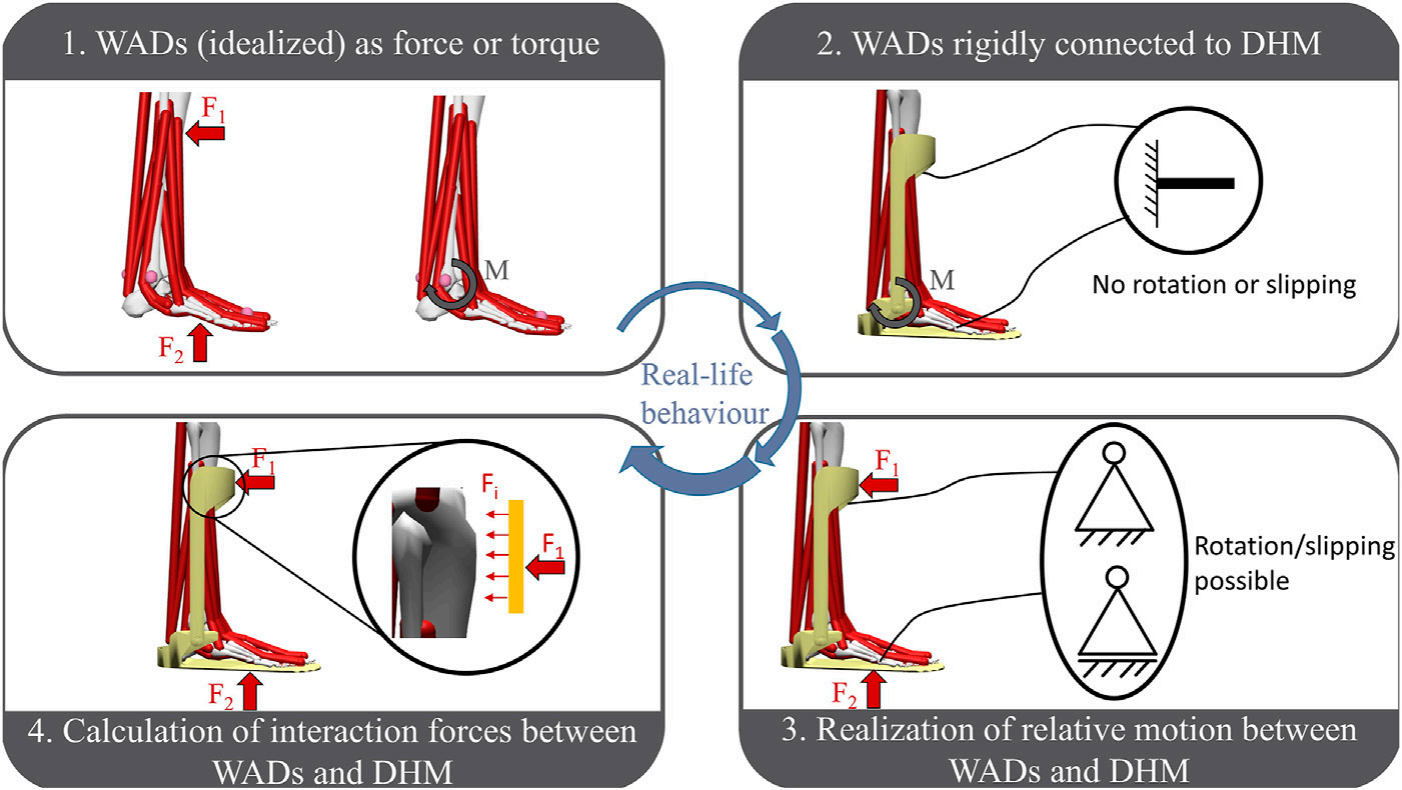
Identified approaches for modelling the interaction between WADs and DHMs arranged according to their reproducibility of the real interface behaviour, represented on the example of an ankle-foot-orthosis (AFO). To symbolise the possible motions at the interface of WAD and AFO figures of mechanical support structures are used.

A detailed list of the included papers and assigned classification for these publications is provided in the Supplementary Material (“Paper classification”). The reviewed literature reveals four different approaches for simultaneously coupling a WAD with a DHM. The approaches are arranged according to their accuracy of reproducing the interaction in the real world. In order to illustrate the used interaction modelling approaches, the example of an ankle-foot orthosis (AFO) is used (Figure 3).

The first group is characterized by the missing virtual representation of the WAD in the simulation environment, which means that only the effect of the device is considered. This effect can either be a provided torque (Cholewicki, 2004; Farris et al., 2014; Karavas et al., 2015; Jackson et al., 2017) or an applied force (Sawicki and Khan, 2016; Inose et al., 2017; Kim et al., 2017; Yang et al., 2019). Furthermore, the efficacy of this assistance is always provided ideally (Uchida et al., 2016; Dembia et al., 2017; Franks et al., 2020). Hence, a torque is provided exactly for the coordinate axis to be supported (Font-Llagunes et al., 2011; Afschrift et al., 2014) or the force is applied to one steady, non-changing point in perfect effective line for the assistance (Ueda et al., 2007; Chen et al., 2019). In this group the abstraction of the WAD’s impact as an added virtual muscle in the DHM accounting for the applied force (Ganesan and Gupta, 2021; Harbauer et al., 2022) is also classified.

In the second group of interaction modelling approaches a virtual representation of the WAD is now considered in the simulation. The interaction of the WAD and the DHM is realized by a rigid connection (Imamura et al., 2011; Ferrati et al., 2013; Lancini et al., 2016) resulting in no possible shift or rotation between the two partners. This connection is done by fixing one point of the device to one point of the model’s corresponding bone (e.g., in the MHM simulation software OpenSim (Delp et al., 2007; Seth et al., 2011; Seth et al., 2018) this is done by a weldJoint (Yamamoto et al., 2021) or *via* a constraint (Ferrati et al., 2013)) and thereby matching the device to the human’s anatomy (see Figure 3.2) (Ferrati et al., 2013; Michaud et al., 2019; Yamamoto et al., 2021). Kruif et al. (2017) even align the WAD to the model’s bones, in other words assumes the device to be integrated into the human kinematical system. In this classification the effect of the inserted WAD is either determined by the set stiffness of the device resulting in a force being applied to the fixation points of device and model (Ferrati et al., 2013; Tröster et al., 2020; Yamamoto et al., 2021) or by applying an external force or torque equally to group 1 (Kruif et al., 2017; Yin et al., 2019; Gordon et al., 2022).

The identified third group for interaction modelling approaches constitutes one basic change compared to the previous one. The virtual representation of the WAD is also present in the simulation environment, but the connection with the model is supplemented by additional degrees of freedom (Figure 3.3) (To et al., 2005; Zhou et al., 2017; Panero et al., 2020). Thereby, translation and rotation of the device to the human model is possible, the execution depends on the studied use case (Arch et al., 2016; Moosavian et al., 2018; Fritzsche et al., 2022). The connection is done *via* kinematic constraints (To et al., 2005; Panero et al., 2019; Liu et al., 2021) or defined joints (e.g., in OpenSim *via* FreeJoint or CustomJoint) (Zhu et al., 2013; Zhou et al., 2015; Arch et al., 2016). Furthermore, the AnyBody modelling software (Damsgaard et al., 2006; AnyBody Technology, 2022) provides a possibility to set the fixed connection between the device and DHM as soft, which allows small relative motions (Zhou et al., 2017; Fritzsche et al., 2022). Due to motion of the device to the model, the force being applied is also not steady and does not operate idealized, which represents the real occurrence during performing with a WAD.

**FIGURE 4:**
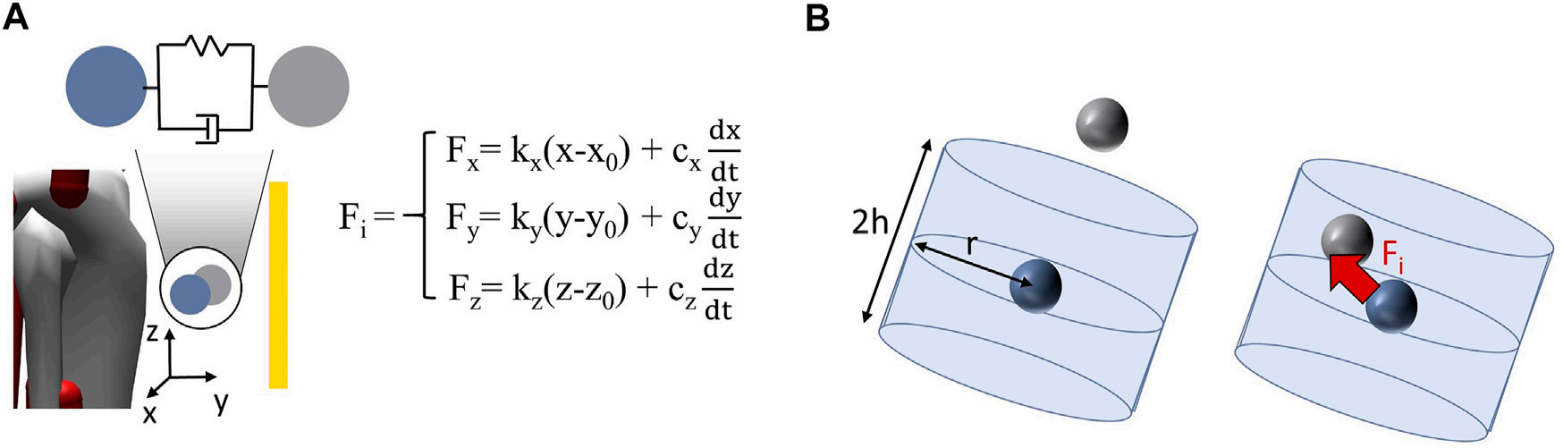
Identified approaches for simulating the occurring interaction force at the interface between DHM und WAD based on the contact models **(A)** based on the deviation of the two contact nodes and the calculation of the force *via* a spring-damper system **(B)** based on the integration of the device node in the cylindrical area around the model node and generation of the force; blue indicates the node of the DHM, grey indicates the node of the WAD.

The fourth classification of interaction modelling approaches between WADs and DHMs highlights no difference to group three in terms of the connection between the two partners. However, an important parameter for the design of the device (Silva et al., 2010; Serrancoli et al., 2019) is considered, which is the occurring interaction force (F_i_ in Figure 4) at the interface. Due to the applied force on the human body by the WADs, the biological soft tissue is on the one hand pressured (pressure force) and on the other hand sheared due to the occuring relative motion (friction force) (Pons, 2010; Silva et al., 2010). Both of these forces combine to the interaction force at the interface of the device and human. The resulting pressure of the interaction over the contact area is a key factor for user comfort, safety and possible occurring injuries (Pons, 2010; Fournier et al., 2018; Serrancoli et al., 2019; Zhang et al., 2021). Therefore, the publications classified in group 4 provide modelling approaches to simulatively estimate the interaction forces and pressures and to evaluate by that the (dis-)comfort of the device. The identified approaches can be divided in two different types, which are both based on using contact models for determining the interaction force. The first type is based on the definition of two points, one for the body and one for the WAD, who are coincident in their initial position (Popovic, 1990; Fournier et al., 2018; Serrancoli et al., 2019; Zhou, 2020). Due to possible relative motion between WAD and DHM, the two points will shift from each other. By applying a tri-directional spring-damper force element between the two anchor points (Lee et al., 2018; Luo et al., 2018; Serrancoli et al., 2019), the occurring interaction force can then be calculated due to the equation shown in Figure 4A (Zhou, 2020). With given stiffness constants k_x_, k_y_, k_z_ and damping constants c_x_, c_y_, c_z_ depending on the resistance in every spatial direction the interaction force in each direction (F_x_, F_y_, F_z_) can be calculated based on the distance of the two points (x, y, z) according to the initial assembly (x_0_, y_0_, z_0_) and the derivative of the distances ( $\frac{dx}{dt},\;\frac{dy}{dt},\;\frac{dz}{dt}$ ) resulting in the interaction force (F_i_)_._ This calculation type accordingly incorporates the viscoelastic behaviour of the soft biological tissue and utilizes the modelling *via* spring and damper. The second type is also based on the definition of one node for each collaborator (Cho et al., 2012; Chander and Cavatorta, 2020; Zhang et al., 2021; Chander et al., 2022). The nodes are in this case not coincident with each other in the initial situation (Figure 4B). The node of the DHM is assigned as the base object and the node of the device as target object (Silva et al., 2010; Cho et al., 2012; Zhang et al., 2021). Then, for the base object a cylindrical shape with pre-given dimensions (height h and radius r) is defined (Cho et al., 2012; Zhang et al., 2021). Contact is assumed, when the node of the device is moved inside the cylinder around the base object, generating the occurring of an interaction force (F_i_). This force is realized by the virtual integration of an artificial muscle, e.g., an actuator in OpenSim (Chander and Cavatorta, 2020). Thus, this type of interaction force calculation is based on a rigid-body method.

## 4 Discussion

**FIGURE 5:**
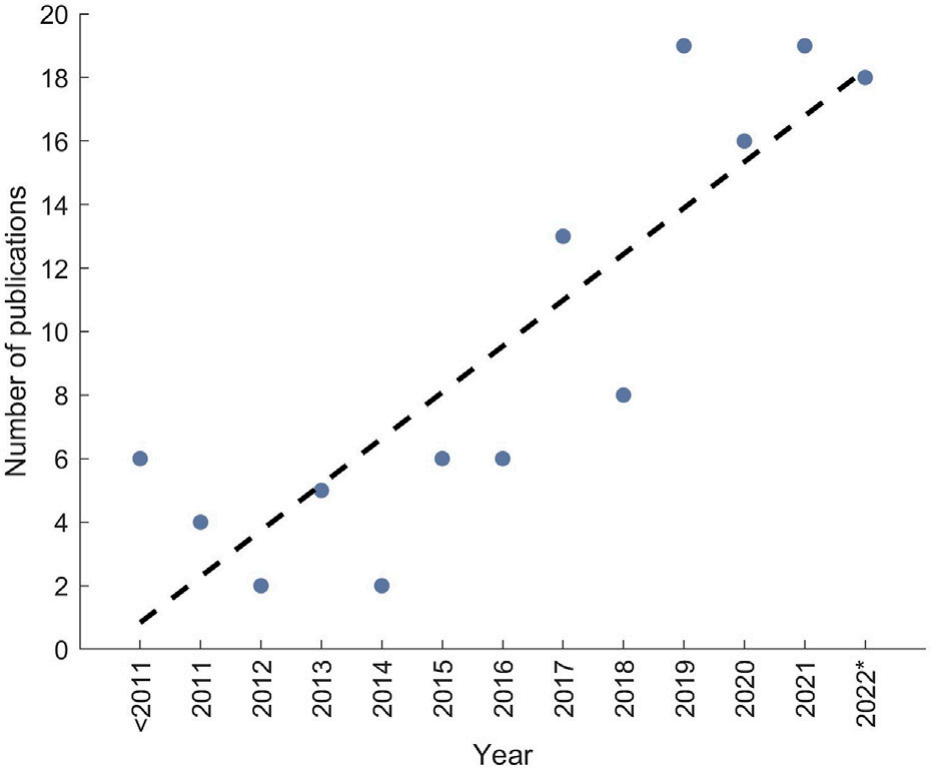
Number of publications arranged according to the year of publication of the included papers to the literature review, the dotted line is the trend of publications; 2022* indicates that literature is only considered that has been published to the date of literature search.

The aim of this systematic review was to identify approaches for modelling the interaction between a WAD and a DHM. The identified approaches should be analysed regarding their consideration of the real-world interface behaviour. Additionally, we wanted to study how this interaction modelling approaches respect the influence of the biological soft tissue at the interface regarding the occurring relative motion between device and human and the effect on the force transmission due to the compliance of biological soft tissue. Arranging the publications included in this literature review by the respective year they were published also shows the increasing use of DHMs for the investigation of the effects of WADs on humans over the last years (Figure 5). Furthermore, this highlights the trend and relevance of this topic for the future design of WADs based more on the findings of virtual results and also the aid that is provided by DHMs to improve the performance and quality of the devices.

### 4.1 Interaction modelling approaches between WADs and DHMS

In this chapter, RQ1 is answered. The systematic review revealed four approaches for modelling the interaction between a WAD and a DHM, which are arranged in ascending order regarding their ability to recreate the occurring interactions in reality. The first level depicts an approach, where only the effect of the device, i.e., force or torque, without any power loss is applied to the model and therefore respected in the simulation. This approach appears as a pretty simplified representation. The force or torque is assumed to operate always in an idealized way and even the device itself is not present in the simulation environment, which also accounts for no possible simulative interaction between the WAD and the model. However, the use of this approach is suitable and beneficial in the early phases of the product design process, like the concept phase, to see and evaluate effects easily and fast (Miehling et al., 2018; Franks et al., 2020; Gneiting et al., 2022). Especially, when the design of the product is not known yet or the treatment of musculoskeletal diseases or their underlying causes is studied, the approximation to the effect of the device is very useful (van der Spek et al., 2003; Bae and Tomizuka, 2011; Bianco et al., 2022). The second approach shows the integration of the WAD in the simulation environment and the coupling of the device with the DHM. By adding the device to the human model, mass, center of mass and inertia is considered (Lancini et al., 2016; Kruif et al., 2017) and accordingly influences the simulation results. The problem of this interaction modelling approach is that device and model are fixed with each other. Thus, no slipping or rotation can occur between the collaborators as in reality. Considering the realisation of rigid fixation of both co-workers, researchers have to be aware of preserving correct kinematic chains. For example, trying to model the shown example of the orthosis (Figure 3- interaction type 2) in OpenSim by applying a weldJoint between both orthosis parts and the corresponding bone (shank and foot) and a rotational joint between both orthosis parts results in a non-viable kinematic chain. A solution could be the use of a constraint as replacement for one weldJoint as done by Kruif et al. (2017) or by combining both parts with other modelling solutions than a joint (Yamamoto et al., 2021). The suitable workaround depends essentially on the use case and WAD that should be modelled. Furthermore, since the connection is rigid, the assistance of the device is still idealized and without the consideration of torque loss as in level one approaches. As a difficult classification, there are some special approaches combining the mass, inertia and center of mass of the WAD with the corresponding bone of the model (Schemschat et al., 2016; Manns et al., 2017; Lerner et al., 2019; Nguyen et al., 2019). Here, a virtual prototype is not present in the simulation, normally accounting for a classification to group one. However, due to the consideration of the devices’ influence on the dynamic simulation results, the publications are classified to level two. Publications classified in level three incorporate the advantages of group two and additionally provide the possibility of relative motions between the device and model. The result is that the assistance is not idealized anymore, but shifts from the optimal effective line, which accounts for a loss of the transmitted torque by device. This behaviour represents the actions also occurring in reality due to misalignment. The relative motion is mainly realized by allowing additional degrees of freedom between WAD and DHM, again having to be aware of viable kinematic chains. In the most publications either one (To et al., 2005; Arch et al., 2016) or two degrees of freedom (Panero et al., 2020; Liu et al., 2021) per fixation point are modelled at most. Due to the kinematic redundancy (Yang et al., 2007) this limitation of degrees of freedom is necessary to allow the model to solve the present problem. The possible degrees of freedom are therefore reduced to the main directions depending on the specific use case. The solution of “soft constraints” implemented in AnyBody depicts another approach to respect the possible relative motions. By setting a constraint to soft, the constraint does not have to be fulfilled, but should be fulfilled as well as possible due to an optimization algorithm (Christensen et al., 2021). During the implementation, the definition of single degrees of freedom in one connection is also possible (like soft for two translations and the third translation is fixed) (Damsgaard et al., 2006). The force from the WAD is then transmitted *via* the resulting, shifting position of the interface. Furthermore, the calculation and determination of the occurring relative motion can also be done in an extra model or tool prior to the DHM (Molz et al., 2022) accounting for a special use case of this level three classification. The force application on the human model is then similar to level one, with the difference that the possible non-ideal effect due to the calculated varying point of force application is considered. Thus, for all solutions a difference in the occurring relative motion from the simulation to the reality is pretty likely, but was not validated in the investigated publications. The identified fourth level introduces and investigates the occurring interaction forces at the interface between WAD and model. By modelling the interaction forces, a virtual user (dis-)comfort assessment is possible. With this gained results, the design of the devices can be improved and optimized to users’ requirements (Fournier et al., 2018; Serrancoli et al., 2019), which further underlines the targeted design of WADs in the virtual environment. However, in some publications only the normal pressure and not the friction or shear forces is investigated, which has an huge influence on the humans’ comfort (Boutwell et al., 2012; Fournier et al., 2018; Zhang et al., 2021). Furthermore, practically no paper did a validation of the simulated interaction force against the real one (Silva et al., 2010; Cho et al., 2012; Zhou, 2020). Serrancoli et al. (2019) were the only ones to compare their predicted forces against experimentally measured ones, but only considered a 2D-movement and did not measure the shear forces, explaining the divergence in force values. Zhang et al. (2021) also point out that rather the trend of the calculated interaction forces should be analysed than the magnitude. Comparing the two different contact models for determining the interaction force, the approach with the force calculation *via* a spring-damper system seems to be the simpler realisation (in terms of modeling/implementation) (Zhou, 2020) by just defining the stiffness and damping constant values in each direction accounting for a linear viscoelastic behaviour. However, a constant value for stiffness and damping constant appears to be questionable due to the resulting, non-linear deformation of the soft biological tissue in reality. The determination of these constant values is also quite a challenge. Furthermore, the interaction force is always calculated, also when the two partners deviate from each other, which means that the WAD is moving away from the body and thus does not produce an interaction force in reality. The second approach with the induction of a force when the device node is in the cylindrical shape around the DHM node is a more modelling-heavy task having to define the nodes of the collaborators, the size and range of the cylindrical shape around the model node and the definition of the artificial muscle. The advantages are the improved definition of the occurring interaction forces (Fournier et al., 2018) and the improved representation of a planar influence on the interaction force (Silva et al., 2010; Cho et al., 2012). On the other hand, the contact force depends on the chosen optimum value of the artificial muscle to activate it. Furthermore, the appearing friction force is also modelled depending on the normal force *via* a friction coefficient assuming a linear, constant dependency between the parameters (Christensen et al., 2021). A comparison of the two calculation types has not been conducted so far. The four interaction modelling types are arranged according to their ability to recreate the occurring interactions in reality. This ranking, however, is only valid in situations, when the included model of the WAD to the DHM is also represented correctly and in good quality. A poor representation or poorly adjusted parameters do not necessarily improve the insights one might gain from the simulation, but rather lead to false implications. More detailed models are not necessarily equivalent to better models. However, the ambition should be directed to realising more complex models, i.e., higher rankings in this classification, in order to account for the transferability of the results to reality but users have to be aware that more uncertainties and accordingly errors can be established. Therefore, a validation of adjusted settings and modifications with the results from real user tests is essential to ensure a high quality of the executed modelling (Serrancoli et al., 2019; Franks et al., 2020).

### 4.2 Consideration of soft biological tissue’s influence at the interface

In the following section, RQ2 is discussed. The influence of the soft biological tissue for limiting the efficiency of the WADs’ assistance to the human body is based on its compliant behaviour. Two main factors/causes for influencing the efficiency resulting from that compliance were identified, the occurring relative motion between device and human—referred to as misalignment—and the influence on the power transmission due to energy absorption. The literature review and the identified interaction modelling approaches show that there are several publications (the ones classified to the previously mentioned group three and four) incorporating the misalignment in their simulation of WADs with DHM. As already mentioned, this implementation is sometimes limited and requires further research, but reached a pretty remarkable advancement in past years. Concerning the energy absorption behaviour of the biological soft tissue and thus affected efficacy of the WAD’s assistance, there are no existing approaches to implement this effect in DHMs. Gordon et al. (2018) are the only ones attempting to integrate the influence in their study. Designing an active pelvis orthosis with a musculoskeletal human model—orthosis fixed to pelvis bone and external torque applied (interaction modelling approach level 2)—the power loss between the generated torque and the experienced torque is simulated. For the generated torque a certain curve was given. The given torque was divided in loading and unloading phase of the human. During loading a percentage of the generated power is absorbed and during unloading a percentage of this absorbed power is returned (Yandell et al., 2017; Young and Ferris, 2017; Sánchez-Villamañán et al., 2019) resulting in a change of the experienced torque curve compared to the generated torque curve. The values of the percentages absorbed and returned were taken from Yandell et al. (2017). The result was that compared to the ideal support of the orthosis qualitative changes in the metabolic energy consumption for different walking conditions and that in these walking conditions (especially for slow walking) strong differences in the energy consumption of single leg muscles, like tibialis posterior or biceps femoris, were observable. However, Gordon et al. (2018) made some assumptions like the simple interaction modelling approach or the pure transfer of the data from Yandell et al. (2017), who determined the absorption and return percentage values on the lower leg. Nevertheless, the results show that a consideration of the soft biological tissue’s influence on the power transmission of WADs in DHMs could have a high benefit and could lead to further improvements for the virtual design of WADs.

### 4.3 Limitations of the conducted systematic literature review

There are also some limitations in our literature review. There is no common term for what we introduced as “wearable assistive devices.” As already mentioned in the introduction, there are many different designations for these types of devices in publications or even just the name of the device itself is mentioned (like exoskeleton or orthosis). The same applies for the “digital human models,” which can be referred to as “musculoskeletal models,” “human models,” “computational models” and so on. This non-uniform nomenclature makes it hard to find a search string including all relevant publications for interaction modelling of WAD and DHM. This problem was assumed to be handled by the development of a rather generic and comprehensive search string capturing a high number of publications and thus, a time-consuming screening process. However, the literature search does not ensure, that all relevant literature was discovered, since only the databases “Scopus” and “Web of Science” were used. To face this limitation on the one hand a further search with the search string in “Google Scholar” was conducted and the first 100 most relevant found literature was studied and on the other hand a backwards search in the reference lists of the identified publications was done to reveal missing relevant publications. Furthermore, a certain bias of the authors in the understanding and interpretation of the described approaches in the literature cannot be eliminated.

## 5 Conclusion and outlook

As a conclusion, in this literature review the approaches for modelling the interaction/interface between WADs and DHMs were investigated. The analyses revealed four different approaches that were introduced and investigated according to their reproduction of the real-life behaviour resulting in a listing of these approaches with increasing accuracy. With a special focus, the consideration of the soft biological tissue’s influence in the modelling approaches was studied. The results show that the occurring relative motion between the device and human—misalignment—in reality is already integrated in the interaction modelling approaches in some way. The influence on the power transmission due to the non-linear viscoelastic behaviour of the soft biological tissue on the other hand is not considered in the modelling approaches so far. Due to the increasing use of DHMs for investigations of the effects of WADs on the human body (compare increasing number of literature in past years in Figure 5), the improvement of the interaction modelling determining the efficiency of the WAD assistance towards the reproduction of the real-life behaviour should be the main vision. By reaching this, the need for time- and cost-consuming user tests will decrease and the quality of the devices can be improved. There are many possible ways to continue existing research, like the comparison between virtual and real relative motion, the validation of simulated and real interaction forces and comparing both identified methods (rigid-body vs. viscoelastic) for simulating the interaction forces. A promising direction, in our opinion, is the development of an approach to consider the soft biological tissue’s influence on the power transmission in the DHM simulation since this is neglected so far. There are mutliple ways to realize such an approach, e.g., by the use of spring-damper-systems (Sánchez-Villamañán et al., 2019) or by combining the tools to compute the interaction force with finite element models (Périé et al., 2004; Cheung and Zhang, 2008). With such a possibility, the device could be better designed according to the natural circumstances and thereby, the efficiency and efficacy of the WADs could be significantly increased.

## Data Availability

The original contributions presented in the study are included in the article/Supplementary Material, further inquiries can be directed to the corresponding author.
