# Supplementary material for: Modelling the interaction between wearable assistive devices and digital human models—A systematic review
Source: Front Bioeng Biotechnol. 2023 Jan 10;10:1044275. doi: 10.3389/fbioe.2022.1044275 (PMC9872199; doi:10.3389/fbioe.2022.1044275)
Supplement: Supplementary file 1 [file DataSheet1.pdf]

Supplementary Material

1 Full search strings

The initial search was conducted on March 18, 2022. A newly check of the literature was conducted August 15, 2022 to identify published data in the meantime.

- Scopus:
  TITLE-ABS-KEY ((("musculoske* model*") OR ("musculoske* simul*") OR ("digital* human model*") OR ("human model*") OR ("biomech* model*") OR ("biomech* simulat*") OR ("computat* model*") OR ("human neurom* model*")) AND ((exoskelet*) OR ("assisti* device*") OR (orthos?s) OR (orthotic*) OR (exosuit*) OR ("assist* robot*") OR ("assist* robot* device*") OR ("support* device*")))
  Search Fields: Title, Abstract and Keywords

- Web of Science:
  ALL=((("musculoske* model*") OR ("musculoske* simul*") OR ("digital* human model*") OR ("human model*") OR ("biomech* model*") OR ("biomech* simulat*") OR ("computat* model*") OR ("human neurom* model*")) AND ((exoskelet*) OR ("assisti* device*") OR (orthos?s) OR (orthotic*) OR (exosuit*) OR ("assist* robot*") OR ("assist* robot* device*") OR ("support* device*")))
  Search Fields: All Fields

Search language = English

2 Exclusion criteria

- Publication not dedicated to the interaction of DHMs and WADs, rather depict the interface between the device and a computer to regulate the device
- DHMs used as a pre-investigating tool for the design of WADs, i.e. biomechanical analysis of diseased humans to gain knowledge for a future design of WAD
- DHMs used to evaluate effects of physical, manufactured WADs on a diseased patient, i.e. WAD not virtually represented and equipped to DHM in simulation

3 Classification of the identified literature

1a) WAD as Torque					
#	Author	Year	Title	WAD	Source
1	Afschrift, M. et al.	2014	The effect of muscle weakness on the capability gap during gross motor function: A simulation study supporting design criteria for exoskeletons of the lower limb	Exoskeleton for lower limb	Scopus
2	Akbas, T. and Sulzer, J.	2019	Musculoskeletal simulation framework for impairment-based exoskeletal assistance post-stroke	Knee exoskeleton	Scopus
3	Alonso, J. et al.	2012	A simple approach to estimate muscle forces and orthosis actuation in powered assisted walking of spinal cord-injured subjects	Lower leg orthosis	Scopus, Web Of Science
4	Bae, J. and Tomizuka, M.	2011	A gait rehabilitation strategy inspired by an iterative learning algorithm	Gait rehabilitation device	Scopus
5	Bianco, N. et al.	2022	Coupled exoskeleton assistance simplifies control and maintains metabolic benefits: A simulation study	Hip, knee and ankle exoskeleton	Scopus, Web Of Science
6	Bianco, N. et al.	2022	Simulated Exoskeletons with Coupled Degrees-of-Freedom Reduce the Metabolic Cost of Walking	Hip, knee and ankle exoskeleton	Scopus
7	Cholewicki, J.	2004	The effects of lumbosacral orthoses on spine stability: What changes in EMG can be expected?	Lumbosacral orthosis	Scopus, Web Of Science
8	Chung, H.-J. et al.	2016	A method of optimization based human dynamic simulation for exoskeleton robot design and assessment	Waist power assist exoskeleton	Scopus
9	Constantinescu, C. et al.	2019	Digital twins of exoskeleton-centered workplaces: Challenges and development methodology	Exoskeletons	Scopus
10	Cseke, B. et al.	2022	Simulating Ideal Assistive Strategies to Reduce the Metabolic Cost of Walking in the Elderly	Hip and ankle exoskeleton	Scopus
11	De Sousa, A.C.C. et al.	2019	Integrating hip exosuit and FES for lower limb rehabilitation in a simulation environment	Hip exosuit	Scopus

12	Dembia, C.L. et al.	2017	Simulating ideal assistive devices to reduce the metabolic cost of walking with heavy loads	Assistive devices	Scopus, Web Of Science
13	Destarac, M.A. et al.	2017	Analysis of the Influence of External Actuators on the Glenohumeral Joint Movements	Shoulder exoskeleton	Scopus
14	Farris, D.J. et al.	2014	Musculoskeletal modelling deconstructs the paradoxical effects of elastic ankle exoskeletons on plantar-flexor mechanics and energetics during hopping	Ankle exoskeleton	Scopus, Web Of Science
15	Font-Lagunes, J.M. et al.	2011	Simulation and design of an active orthosis for an incomplete spinal cord injured subject	Lower leg orthosis	Scopus
16	Franks, P.W. et al.	2020	Testing Simulated Assistance Strategies on a Hip-Knee-Ankle Exoskeleton: A Case Study	Hip, knee and ankle exoskeleton	Scopus
17	Gallagher, W. et al.	2013	Relaxed individual control of skeletal muscle forces via physical human-robot interaction	Arm exoskeleton	Scopus, Web Of Science
18	Gneiting, E. et al.	2022	Model-Based Biomechanics for Conceptual Exoskeleton Support Estimation Applied for a Lifting Task	Arm exoskeleton	Scopus
19	Han, J.I. et al.	2020	Simulation of a lower extremity assistive device for resistance training in a microgravity environment	Hip, knee and ankle rehabilitation device	Scopus, Web Of Science
20	Hegarty, A.K. et al.	2017	Evaluating the effects of ankle-foot orthosis mechanical property assumptions on gait simulation muscle force results	Ankle-foot orthosis	Scopus, Web Of Science
21	Hussain, S. et al.	2013	Effect of cadence regulation on muscle activation patterns during robot-assisted gait: A dynamic simulation study	Lower leg assistance robot	Scopus, Web Of Science
22	Ippolito, D. et al.	2020	Enhancement of human-centered workplace design and optimization with Exoskeleton technology	Exoskeletons	Scopus
23	Jackson, R.W. et al.	2017	Muscle-tendon mechanics explain unexpected effects of exoskeleton assistance on metabolic rate during walking	Ankle exoskeleton	Scopus, Web Of Science
24	Karavas, N. et al.	2015	Tele-impedance based assistive control for a compliant knee exoskeleton	Knee exoskeleton	Scopus, Web Of Science
25	Li, N. et al.	2020	Bioinspired Musculoskeletal Model-based Soft Wrist Exoskeleton for Stroke Rehabilitation	Wrist exoskeleton	Scopus, Web Of Science

26	Liu, Y. et al.	2021	A Home-Based Bilateral Rehabilitation System with sEMG-based Real-Time Variable Stiffness	Arm rehabilitation device	Scopus, Web Of Science
27	McLain, B.J. et al.	2022	Effect of Assistance Using a Bilateral Robotic Knee Exoskeleton on Tibiofemoral Force Using a Neuromuscular Model	Knee exosksleton	Scopus, Web Of Science
28	Miehling, J. et al.	2018	Musculoskeletal simulation and evaluation of support system designs	Knee support system	Scopus
29	Mghames, S. et al.	2019	A neuromuscular-model based control strategy to minimize muscle effort in assistive exoskeletons	Arm exoskeleton	Scopus
30	Qiu, S. et al.	2020	Conditions for active assistance control of exoskeleton robot	Hip exoskeleton	Scopus
31	Ren, B. et al.	2022	Gait trajectory-based interactive controller for lower limb exoskeletons for construction workers	Lower limb exoskeleton	Scopus, Web Of Science
32	Seyfarth, A. et al.	2015	Soft Robotics: Transferring Theory to Application	Soft robotics	Scopus
33	Sato, M. et al.	2011	A study on power assist suit using pneumatic actuators based on calculated retaining torques for lift-up motion	Power assist suit	Scopus
34	Stollenmaier, M. et al.	2020	Simulating the response of a neuro-musculoskeletal model to assistive forces: Implications for the design of wearables compensating for motor control deficits	Arm assistive devices	Scopus
35	Uchida, T. et al.	2016	Simulating ideal assistive devices to reduce the metabolic cost of running	Assistive devices	Backwards search
36	Wang, N. et al.	2021	Assistive Torque of Ankle Exoskeleton Based on a Simple Biomechanical Model and a Genetic Algorithm	Ankle exoskeleton	Scopus
37	Willso, A.M. et al.	2020	Design and development of a quasi- passive transtibial biarticular prosthesis to replicate gastrocnemius function in walking	Transtibial prothesis	Scopus
38	Zhang, F. et al.	2021	Optimization of Gait Assistance Pattern for Charcot-Marie-Tooth Patients Based on Forward Predictive Simulation	Hip, knee and ankle orthosis	Scopus
39	Zhao, Y.	2017	Kinematics and dynamics analysis of exoskeleton training robot based on the human biomechanical model	Lower leg exoskeleton	Scopus

1b) WAD as idealized force					
#	Author	Year	Title	WAD	Source
1	Chen, W. et al.	2019	On the biological mechanics and energetics of the hip joint muscle-tendon system assisted by passive hip exoskeleton	Hip exoskeleton	Scopus, Web Of Science
2	De Sousa, A.C.C. et al.	2021	Passive Knee Orthoses Assistance in Functional Electrical Stimulation Cycling in an Individual with Spinal Cord Injury	Knee orthosis	Scopus, Web Of Science
3	Ganesan, K. and Gupta, A.	2021	Human-Centric Optimal Design of Biomimetic Exosuit for Loaded Walking: A Simulation Study	Exosuit	Scopus
4	Gneiting, E. et al.	2022	Model-Based Biomechanics for Conceptual Exoskeleton Support Estimation Applied for a Lifting Task	Arm exoskeleton	Scopus
5	Guo, W. et al.	2018	Research on Passive Assist of Hip Joint Based on Muscle Metabolism	Hip exoskeleton	Scopus
6	Harbauer, C..M. et al.	2022	Optimizing Force Transfer in a Soft Exoskeleton Using Biomechanical Modeling	Soft arm exoskeleton	Scopus
7	Van der Have, A. et al.	2022	The Exo4Work shoulder exoskeleton effectively reduces muscle and joint loading during simulated occupational tasks above shoulder height	Shoulder exoskeleton	Scopus, Web Of Science
8	Hu, B. et al.	2021	Design and assist-as-needed control of flexible elbow exoskeleton actuated by nonlinear series elastic cable driven mechanism	Elbow exoskeleton	Scopus, Web Of Science
9	Inose, H. et al.	2017	Semi-endoskeleton-type waist assist AB-wear suit equipped with compressive force reduction mechanism	Upper limb exoskeleton	Scopus
10	Kim, S. et al.	2017	Analysis of Finger Muscular Forces using a Wearable Hand Exoskeleton System	Hand exoskeleton	Scopus, Web Of Science
11	Lee, J. et al.	2015	Design of a hand exoskeleton for biomechanical analysis of the stroke hand	Hand exoskeleton	Scopus
12	Moosavian, S. et al.	2021	RoboWalk: augmented human-robot mathematical modelling for design optimization	Lower limb exoskeleton	Scopus, Web Of Science

13	Nabipour, M. et al.	2021	Human model in the loop design optimization for RoboWalk wearable device	Lower limb exoskeleton	Scopus, Web Of Science
14	Rivera, F.G. et al.	2021	A Framework to Model the Use of Exoskeletons in DHM Tools	Upper limb exoskeleton	Scopus
15	Swaicki, G.S. et al.	2016	A Simple Model to Estimate Plantarflexor Muscle-Tendon Mechanics and Energetics During Walking With Elastic Ankle Exoskeletons	Ankle exoskeleton	Scopus, Web Of Science
16	Triolo, R. et al.	2001	Effects of stimulated hip extension moment and position on upper-limb support forces during FNS-induced standing - A technical note	Lower leg exoskeleton	Scopus, Web Of Science
17	Ueda, J. et al.	2017	Pinpointed control of muscles by using power-assisting device	Arm exoskeleton	Scopus
18	Van der Have, A. et al.	2022	The Exo4Work shoulder exoskeleton effectively reduces muscle and joint loading during simulated occupational tasks above shoulder height	Shoulder exoskeleton	Scopus, Web Of Science
19	Van der Spek, J.H. et al.	2003	A Model-Based Approach to Stabilizing Crutch Supported Paraplegic Standing by Artificial Hip Joint Stiffness	Hip exoskeleton	Scopus, Web Of Science
20	Wu, Y. et al.	2019	Biomechanical simulation analysis of human lower limbs assisted by exoskeleton	Lower limb exoskeleton	Scopus
21	Yang, X. et al.	2019	Spine-Inspired Continuum Soft Exoskeleton for Stoop Lifting Assistance	Back exoskeleton	Scopus, Web Of Science
22	Zhang, X. et al.	2020	A Control Strategy for Maintaining Gait Stability and Reducing Body-Exoskeleton Interference Force in Load-Carrying Exoskeleton	Upper limb exoskeleton	Scopus, Web Of Science
23	Zignoli, A. et al.	2019	Including a Musculoskeletal Model in the Control Loop of an Assistive Robot for the Design of Optimal Target Forces	Assistive Robot	Scopus
2) WAD rigidly connected to DHM					
#	Author	Year	Title	WAD	Source
1	Aliman, N. et al.	2018	Modeling and co-simulation of actuator control for lower limb exoskeleton	Lower limb exoskeleton	Scopus, Web Of Science

2	Ashari, M.F. et al.	2022	Evaluation of Upper Limb Muscle Activation Using Musculoskeletal Model with Wearable Assistive Device	Upper limb assistive device	Scopus, Web Of Science
3	Chengxin, Y. et al.	2020	Investigation of human-device interaction via predictive simulation	Arm exoskeleton	Scopus
4	De Kruif, B.J. et al.	2017	Simulation Architecture for Modelling Interaction Between User and Elbow-articulated Exoskeleton	Elbow exoskeleton	Scopus, Web Of Science
5	Ferrati, F. et al.	2013	Virtual modelling of a real exoskeleton constrained to a human musculoskeletal model	Lower limb exoskeleton	Scopus
6	Gordon, D.F.N. et al.	2022	Human-in-the-Loop Optimization of Exoskeleton Assistance Via Online Simulation of Metabolic Cost	Pelvis exoskeleton	Scopus, Web Of Science
7	Gordon, D.F.N. et al.	2018	Effectively quantifying the performance of lower-limb exoskeletons over a range of walking conditions	Pelvis exoskeleton	Scopus
8	Imamura, Y. et al.	2011	Motion-based-design of elastic material for passive assistive device using musculoskeletal model	Upper body exosuit	Scopus
9	Lancini, M. et al.	2016	Healthcare Sensor System Exploiting Instrumented Crutches for Force Measurement during Assisted Gait of Exoskeleton Users	Lower limb exoskeleton	Scopus, Web Of Science
10	Lerner, Z.F. et al.	2019	Computational modeling of neuromuscular response to swing-phase robotic knee extension assistance in cerebral palsy	Knee exoskeleton	Scopus, Web Of Science
11	Luo, R. et al.	2021	Simulation of Assisted Human Walking Using Musculoskeletal Model Coupled with Exoskeleton via Deep Reinforcement Learning	Lower limb exoskeleton	Scopus
12	Manns, P. et al.	2017	Motion Optimization and Parameter Identification for a Human and Lower Back Exoskeleton Model	Back exoskeleton	Scopus, Web Of Science
13	Michaud, F. et al.	2019	Energy expenditure estimation during crutch-orthosis-assisted gait of a spinal-cord-injured subject	Lower limb exoskeleton	Scopus, Web Of Science
14	Nguyen, V. et al.	2019	Predictive simulation of human walking augmented by a powered ankle exoskeleton	Ankle exoskeleton	Scopus
15	Sambhav, R. et al.	2022	An Integrated Dynamic Closed Loop Simulation Platform for Elbow Flexion Augmentation Using an Upper Limb Exosuit Model	Elbow exosuit	Scopus

16	Schemschat, R.M. et al.	2017	Optimization based analysis of push recovery during walking motions to support the design of lower-limb exoskeletons	Lower limb exoskeleton	Scopus
17	Shi, L. et al.	2018	Model-based active impedance controller development of the exoskeleton rehabilitation robot (ERRobot) for lower-extremity	Lower limb exoskeleton	Scopus
18	Tröster, M. et al.	2020	Biomechanical model-based development of an active occupational upper-limb exoskeleton to support healthcare workers in the surgery waiting room	Shoulder exoskeleton	Scopus, Web Of Science
19	Vieira, H. et al.	2019	Study of the risk of ankle injury during impact on the ground and definition of support orthoses	Ankle-foot orthosis	Scopus
20	Wang, Y. et al.	2021	Simulation design of flexible unpowerd lower limb exoskeleton	Lower limb exoskeleton	Scopus
21	Xu, J. et al.	2019	Design and Implementation of the Lower Extremity Robotic Exoskeleton with Magnetorheological Actuators	Lower leg exoskeleton	Scopus
22	Yamamoto, M. et al.	2019	Effect of an ankle–foot orthosis on gait kinematics and kinetics: case study of post-stroke gait using a musculoskeletal model and an orthosis model	Ankle-foot orthosis	Scopus, Web Of Science
23	Yin, K. et al.	2019	Personalised Control of Robotic Ankle Exoskeleton Through Experience-Based Adaptive Fuzzy Inference	Ankle exoskeleton	Scopus, Web Of Science
3) Realization of relative motion between WAD and DHM					
#	Author	Year	Title	WAD	Source
1	Agarwal, P. et al.	2013	A novel framework for virtual prototyping of rehabilitation exoskeletons	Finger rehabilitation device	Scopus
2	Arch, E.S. et al.	2016	Passive-dynamic ankle-foot orthosis replicates soleus but not gastrocnemius muscle function during stance in gait: Insights for orthosis prescription	Ankle-foot orthosis	Scopus, Web Of Science
3	Arslan, Y.Z. et al.	2019	Exoskeletons, Exomusculatures, Exosuits: Dynamic Modeling and Simulation	Exoskeletons, Exosuits	Scopus
4	Delgado, P. et al.	2022	Human-Exoskeleton Joint Coordination Assessment: A Case Study on the Shoulder and Elbow Joints	Upper limb exoskeleton	Scopus, Web Of Science

5	Fritzsche, L. et al.	2021	Assessing the efficiency of exoskeletons in physical strain reduction by biomechanical simulation with AnyBody Modeling System	Shoulder exoskeleton	Scopus
6	Fritzsche, L. et al.	2022	Assessing the Efficiency of Industrial Exoskeletons with Biomechanical Modelling – Comparison of Experimental and Simulation Results	Shoulder exoskeleton	Backwards search
7	Liu, Y.-X. et al.	2021	Weight Distribution of a Knee Exoskeleton Influences Muscle Activities during Movements	Knee exoskeleton	Scopus, Web Of Science
8	Millard, M. et al.	2017	Predicting the motions and forces of wearable robotic systems using optimal control	Exoskeleton	Scopus
9	Mohamadi, M.R. et al.	2019	Simulation Analysis of Human-RoboWalk Augmented Model	Lower limb exoskeleton	Scopus
10	Molz, C. et al.	2022	A Musculoskeletal Human Model-Based Approach for Evaluating Support Concepts of Exoskeletons for Selected Use Cases	Upper body exoskeleton	Scopus
11	Moon, C. et al.	2022	A Lower-Back Exoskeleton with a Four-Bar Linkage Structure for Providing Extensor Moment and Lumbar Traction Force	Lower back exoskeleton	Scopus, Web Of Science
12	Moosavian, S.A.A. et al.	2019	Augmented Modeling of a Lower Limb Assistant Robot and Human Body	Lower limb exoskeleton	Scopus
13	Panero, E. et al.	2020	Multibody Analysis of a 3D Human Model with Trunk Exoskeleton for Industrial Applications	Trunk exoskeleton	Scopus
14	Panero, E. et al.	2019	Influence of hinge positioning on human joint torque in industrial trunk exoskeleton	Trunk exoskeleton	Scopus
15	Rose, L. et al.	2022	A model-free deep reinforcement learning approach for control of exoskeleton gait patterns	Lower limb exoskeleton	Scopus, Web Of Science
16	Song, J. et al.	2021	Multijoint passive elastic spine exoskeleton for stoop lifting assistance	Spine exoskeleton	Scopus, Web Of Science
17	To, C.S. et al.	2005	Simulation of a functional neuromuscular stimulation powered mechanical gait orthosis with coordinated joint locking	Gait orthosis	Scopus, Web Of Science
18	Zhou, L. et al.	2015	Modeling and design of a spring-loaded, cable-driven, wearable exoskeleton for the upper extremity	Arm exoskeleton	Scopus, Web Of Science

19	Zhou, L. et al.	2016	Design optimization on passive exoskeletons through musculoskeletal model simulation	Arm exoskeleton	Scopus
20	Zhou, L. et al.	2017	A human-centered design optimization approach for robotic exoskeletons through biomechanical simulation	Arm exoskeleton	Scopus, Web Of Science
21	Zhu, Y. et al.	2013	Biomimetic design and biomechanical simulation of a 15-DOF lower extremity exoskeleton	Lower limb exoskeleton	Scopus
22	Zhu, Y. et al.	2015	Biomechanical modeling and load-carrying simulation of lower limb exoskeleton	Lower limb exoskeleton	Scopus, Web Of Science
23	Zoccali, A. et al.	2021	Comfort perception analysis of human models interfacing with novel biped-wheeled-exoskeletons	Whole body exoskeleton	Scopus

4a) Calculation of interaction forces between WADs and DHM via contact modeling

#	Author	Year	Title	WAD	Source
1	Chander, D.S. and Cavatorta, M.P.	2020	Modelling interaction forces at a curved physical human-exoskeleton interface	Lower limb exoskeleton	Scopus
2	Chander, D.S. et al.	2022	Simulating the Dynamics of a Human-Exoskeleton System Using Kinematic Data with Misalignment Between the Human and Exoskeleton Joints	Lower limb exoskeleton	Scopus
3	Cho, K. et al.	2012	Analysis and evaluation of a combined human-exoskeleton model under two different constraints condition	Upper limb exoskeleton	Backwards search
4	Christensen, S et al.	2021	Modeling and Analysis of Physical Human-Robot Interaction of an Upper Body Exoskeleton in Assistive Applications	Upper body exoskeleton	Scopus, Web Of Science
5	Fournier, B.N. et al.	2018	Modeling and Simulation of a Lower Extremity Powered Exoskeleton	Lower limb exoskeleton	Scopus, Web Of Science
6	Jung, M. et al.	2017	Musculoskeletal simulation of SOLEUS ankle exoskeleton for countermeasure exercise in space	Ankle exoskeleton	Scopus
7	Silva, P.C. et al.	2010	Evaluation of the contact forces developed in the lower limb/orthosis interface for comfort design	Ankle-foot orthosis	Scopus, Web Of Science

8	Sreenivasa, M. et al.	2017	Optimizing Wearable Assistive Devices with Neuromuscular Models and Optimal Control	Upper body exoskeleton	Scopus
9	Tröster, M. et al.	2022	Biomechanical Analysis of Stoop and Free-Style Squat Lifting and Lowering with a Generic Back-Support Exoskeleton Model	Back exoskeleton	Scopus, Web Of Science
10	Zhang, L. et al.	2021	Modeling and Simulation of a Human Knee Exoskeleton's Assistive Strategies and Interaction	Knee exoskeleton	Scopus, Web Of Science
4b) Calculation of interaction forces between WADs and DHM via spring-damper-system					
#	Author	Year	Title	WAD	Source
1	Lee, H. et al.	2018	Control of a nonanthropomorphic exoskeleton for multi-joint assistance by contact force generation	Lower limb exoskeleton	Scopus, Web Of Science
2	Luo, R. et al.	2018	Adaptive CPG-Based Impedance Control for Assistive Lower Limb Exoskeleton	Lower limb exoskeleton	Scopus
3	Popovic, D.B.	1990	Dynamics of the self-fitting modular orthosis	Lower limb orthosis	Scopus
4	Serrancoli, G. et al.	2019	Subject-Exoskeleton Contact Model Calibration Leads to Accurate Interaction Force Predictions	Lower limb exoskeleton	Scopus, Web Of Science
5	Wang, Y. et al.	2020	A Coupling Dynamic Model for Studying the Physical Interaction between a Finger Exoskeleton and a Human Finger	Finger exoskeleton	Scopus, Web Of Science
6	Zhou, X.	2020	Predictive human-in-the-loop simulations for assistive exoskeletons	Exoskeletons	Scopus
7	Zhou, X. and Chen, X.	2021	Design and evaluation of torque compensation controllers for a lower extremity exoskeleton	Lower limb exoskeleton	Scopus, Web Of Science
8	Zhou, X. et al.	2020	Evaluation of a 1-DOF Hand Exoskeleton for Neuromuscular Rehabilitation	Hand exoskeleton	Scopus
